# Identification of cold stress responsive microRNAs in two winter turnip rape (*Brassica rapa* L.) by high throughput sequencing

**DOI:** 10.1186/s12870-018-1242-4

**Published:** 2018-03-27

**Authors:** Xiucun Zeng, Yaozhao Xu, Jinjin Jiang, Fenqin Zhang, Li Ma, Dewei Wu, Youping Wang, Wancang Sun

**Affiliations:** 1 College of Agronomy and Biotechnology, Hexi University, Zhangye, 734000 China; 2 College of Agronomy, Gansu Agricultural University, Lanzhou, 730070 China; 3 Jiangsu Provincial Key Laboratory of Crop Genetics and Physiology, Yangzhou University, Yangzhou, 225009 China

**Keywords:** *Brassica rapa*, Turnip, microRNA, Cold stress, Target gene

## Abstract

**Background:**

Low temperature is a major abiotic stress affecting the production of rapeseed in China by impeding plant growth and development. A comprehensive knowledge of small-RNA expression pattern in *Brassica rapa* under cold stress could improve our knowledge of microRNA-mediated stress responses.

**Results:**

A total of 353 cold-responsive miRNAs, 84 putative novel and 269 conserved miRNAs, were identified from the leaves and roots of two winter turnip rape varieties ‘Longyou 7’ (cold-tolerant) and ‘Tianyou 4’ (cold-sensitive), which were stressed under − 4 °C for 8 h. Eight conserved (miR166h-3p-1, miR398b-3p, miR398b-3p-1, miR408d, miR156a-5p, miR396h, miR845a-1, miR166u) and two novel miRNAs (Bra-novel-miR3153-5p and Bra-novel-miR3172-5p) were differentially expressed in leaves of ‘Longyou 7’ under cold stress. Bra-novel-miR3936-5p was up-regulated in roots of ‘Longyou 7’ under cold stress. Four and five conserved miRNAs were differentially expressed in leaves and roots of ‘Tianyou 4’ after cold stress. Besides, we found two conserved miRNAs (miR319e and miR166m-2) were down-regulated in non-stressed roots of ‘Longyou 7’ compared with ‘Tianyou 4’. After cold stress, we found two and eight miRNAs were differentially expressed in leaves and roots of ‘Longyou 7’ compared with ‘Tianyou 4’. The differentially expressed miRNAs between two cultivars under cold stress include novel miRNAs and the members of the miR166 and miR319 families. A total of 211 target genes for 15 known miRNAs and two novel miRNAs were predicted by bioinformatic analysis, mainly involved in metabolic processes and stress responses. Five differentially expressed miRNAs and predicted target genes were confirmed by quantitative reverse transcription PCR, and the expressional changes of target genes were negatively correlated to differentially expressed miRNAs. Our data indicated that some candidate miRNAs (e.g., miR166e, miR319, and Bra-novel-miR3936-5p) may play important roles in plant response to cold stress.

**Conclusions:**

Our work indicates that miRNA and putative target genes mediated metabolic processes and stress responses are significant to cold tolerance in *B. rapa*.

**Electronic supplementary material:**

The online version of this article (10.1186/s12870-018-1242-4) contains supplementary material, which is available to authorized users.

## Background

Cold tolerance is a survival strategy of some overwintering plants in frigid winter. Cold-tolerant plants respond and adapt to cold stress through a number of biochemical changes, such as accumulation of metabolites [1, 2], synthesis of unsaturated fatty acids [3], production of antifreeze proteins [4], and increase of heat shock proteins [5]. In response to cold stress, plants have established stress-responsive molecular regulation mechanisms at the transcriptional, translational, and post-translational levels [6]. Plant cells perceive cold stress signals by activating specific transcription factors (TFs) and a series of complex signal transductions. Subsequently, the TFs combine with *cis*-acting elements to promote and regulate the transcription and expression of target genes, thereby improving the cold-tolerance capability of plants [7, 8].

MicroRNAs have been recently reported as new regulator in plant adaptation to environmental stresses [9, 10], which are small (20–24 nt), endogenous, and noncoding RNAs originate from 70 to 80 nt pre-miRNAs with stem-loop structures. Pre-miRNAs are sequentially processed by Dicer-like proteins in cell nucleus [11] and then transferred to cytoplasm before assembled to RNA-induced silencing complexes (RISCs), which combine with Argonaute proteins. RISCs negatively regulate target gene expression by undergoing base pairing with the 3′ UTR and coding regions of mRNA [12, 13], inducing the degradation of target mRNA or inhibition of translation [14–16]. Therefore, miRNAs play a vital role in regulating gene expression [17, 18]. Numerous studies concentrated on their role in plant response to various stresses have been reported [7, 8]. To date, stress-responsive miRNAs in a diverse number of plants under adverse conditions were reported [19, 20]. The first plant defense miRNA discovered was miR393 in *Arabidopsis* [21, 22], which could increase the amount of cold-induced proteins by depressing its target gene (*ubiquitin E3 ligase*) [23, 24]. Furthermore, many miRNAs related to cold tolerance, such as miR156, miR166, miR172,miR319, miR396, and miR397, are identified in plants with capability of regulating cold stress responses [24–27]. Presently, miRNA related to cold stress is in need to be identified in *Brassica*, which is of great economic value in edible vegetable and oil production.

Winter turnip rape (*B. rapa* L.) is a valuable oil crop for its high-quality edible oil in northwestern China, which is also capable of conserving soil and water in winter and spring [28]. However, extremely low temperature negatively affects the growth and development of winter turnip rape, which consequently fails to overwinter and propagate in northwestern China. The aboveground parts of winter turnip rape are withered during winter, whereas the roots are capable of overwintering, indicating the root tolerance to cold is important for its survival in winter [29]. Unlike *Arabidopsis* and other plants, cold-tolerance in winter turnip rape is rarely studied. ‘Longyou 7’ is the first cultivated cold-tolerant winter turnip rape variety that can survive at extremely low temperatures (− 32 °C, overwinter survival rate is more than 90%). And ‘Tianyou 4’ is a cold-sensitive winter turnip rape variety. Recently, our group has investigated the physiological, biochemical, and molecular mechanisms of cold-tolerance in winter turnip rape [28–31]. Whereas the miRNAs and target genes with putative functions in cold tolerance of *B. rapa* has not been reported yet. In the present study, we constructed and sequenced small RNA (sRNA) libraries from leaves and roots of two winter turnip rape varieties (‘Longyou 7’ and ‘Tianyou 4’) treated with cold stress (− 4 °C), to acknowledge the expressional differences of miRNAs related to cold stress in *B. rapa*. The miRNAs and target genes identified in this study could facilitate the understanding of molecular mechanisms of cold stress signaling in *B. rapa*.

## Methods

### Plant materials, cold stress treatment and physiological analysis

Winter turnip rape ‘Longyou 7’ (cold-tolerant cultivar) and ‘Tianyou 4’ (cold-sensitive cultivar) were provided by the College of Agriculture, Gansu Agricultural University (Lanzhou, China). Plants were grown in plastic pots until six true leaves in a ventilated greenhouse (20 °C, 16 h light/8 h dark cycle), and then moved into growth chamber (Safu, Ningbo, China) for treatment. Cold treatment was started by gradually decreasing the temperature from 20 °C to − 4 °C. The plants were grown at 20 °C for 48 h and then induced at 10 °C and 4 °C for 48 h, respectively. Finally, they were stressed at − 4 °C for 8 h. The leaves and roots of plants grown at 20 °C (as control, CK) and − 4 °C (as treatments, TR) were collected and frozen in liquid nitrogen for RNA extraction and physiological analysis. Generally, eight samples were collected and named 4LCK (leaf of ‘Tianyou 4’ at 20 °C), 4LTR (leaf of ‘Tianyou 4’ treated at − 4 °C), 4RCK (root of ‘Tianyou 4’ at 20 °C), 4RTR (root of ‘Tianyou 4’ treated at − 4 °C), 7LCK (leaf of ‘Longyou 7’ at 20 °C), 7LTR (leaf of ‘Longyou 7’ treated at − 4 °C), 7RCK (root of ‘Longyou 7’ at 20 °C), and 7RTR (root of ‘Longyou 7’ treated at − 4 °C), respectively. Each sample were polled from three plants and two biological replicates were included. Malondialdehyde (MDA) content and peroxidase (POD) activities was analyzed according to Campos et al. [32] and Quiroga et al. [33].

### RNA isolation, library construction and sequencing

Total RNA was extracted using TRNzol Universal Reagent (Tiangen, China) following the manufacturer’s instructions. The quality and concentration of RNA were tested with Agilent 2100 bio-analyzer. The sRNA libraries were constructed as previously described [34, 35]. In brief, the 18–30 nt sRNAs of each sample were gel purified from the total RNA. Two adaptors (5′ and 3′ adaptors) were ligated to the purified sRNAs successively and then reversely transcribed and amplified for 15 cycles by PCR. Then sRNA libraries (including two biological replicates) of the eight samples were constructed. The quality and production of the libraries were further tested by Agilent 2100 Bioanalyzer and ABI StepOnePlus Real-Time PCR system, and then sequenced on an Illumina Genome Analyzer by the Beijing Genomics Institute in Shenzhen (http://www.genomics.cn/en/index).

### Analysis of small RNA sequencing data

Raw data from high-throughput sequencing were filtered as previously described to obtain clean reads [35, 36]. The process included the removal of adapters and low-quality sequences containing poly A and less than 18 nucleotides. Next, the clean sequences were mapped onto the *B. rapa* genome and Rfam12.2 (http://rfam.xfam.org/) to remove noncoding RNAs, such as rRNAs, tRNAs, snRNAs, and snoRNA. The obtained sequences after screening were searched in the miRBase 21.0 database (http://www.mirbase.org) to identify known mature miRNAs. The sRNAs matched to known plant miRNAs with fewer than three mismatches, and could form typical stem-loop secondary structures, were identified as conserved miRNAs [37]. Sequences that were unmapped to any miRNA in the miRBase and had less than two mismatches were taken as novel miRNAs by miRDeep2 (https://www.mdc-berlin.de/8551903/en).

### Differential expression analysis of miRNAs

miRNA expression level was calculated by Transcripts Per Kilobase Million (TPM), which can eliminate sequencing discrepancy [38]. Differential expression of miRNA between two samples was analyzed by DESeq2 [39, 40]. miRNAs were considered up-regulated when the fold change was greater than 2 or down-regulated when the Padj value (adjusted *p*-value) was less than 0.01. TPM and fold change were calculated as previously described [41].

### Target gene predication and enrichment analysis

Target gene prediction of miRNAs was analyzed by psRobot (http://omicslab.genetics.ac.cn/psRobot) and TargetFinder (https://github.com/carringtonlab/TargetFinder) according to the related references [42, 43]. The targets of known and novel miRNAs with different expression between two samples were subjected to Gene Ontology (GO) annotations using the WEGO package [44].

### Expression analysis of miRNAs and predicted target genes using qRT-PCR

Stem-loop quantitative reverse transcription PCR (qRT-PCR) was performed to analyze miRNA expression. According to the mature miRNA sequence, the specific stem-loop reverse transcription (RT) primers and forward primers of seven selected miRNAs were designed following the method described by Chen et al. [45]. The universal reverse primer was used for all qRT-PCR reactions and U6 snRNA was used as an endogenous reference gene. For qRT-PCR validation of target gene, first-strand cDNA was generated using a SuperScript®III RT Reagent Kit (Invitrogen, China, Beijing). The *Actin of B. rapa* was used as an endogenous reference gene for normalization. The primer sequences for validation of miRNA and target gene expression are listed in Additional file 1: Table S1 and Additional file 2: Table S2, respectively. qRT-PCR analysis was carried out on 7900HT Fast Real-Time PCR System (Applied Biosystems, USA) using SYBR qPCR Mix (Invitrogen). The PCR conditions were 95 °C for 2 min, followed by 40 cycles of 94 °C for 20 s, 60 °C for 20 s, and 72 °C for 30 s. Triplicate technical replicates were run for qPCR, and amplification products were further confirmed by analyzing the melting curve and gel electrophoresis. The relative expression of miRNAs was calculated using the 2^−ΔΔCt^ method [46].

## Results

### Physiological responses to cold stress

**Fig. 1:**
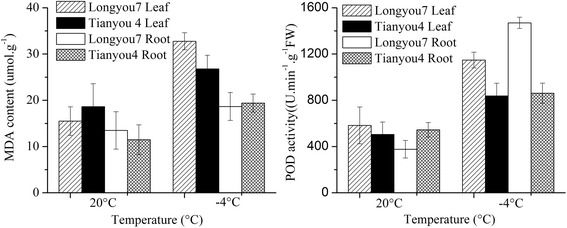
The responses of MDA content and POD activity to cold stress

The leaf and root tissues of ‘Longyou 7’ and ‘Tianyou 4’ were used to study cold responses. The degree of cold tolerance was estimated by measuring MDA content and POD activity, which were significantly increased in the leaves and roots of the two varieties under cold stress (Fig. 1). We found the MDA content and POD activity with no significant difference between two cultivars grown at 20 °C. However, the MDA content of 7LTR was higher than that of 4LTR. No significant difference of MDA content was found between the cold stressed roots. POD activity in the leaves and roots of ‘Longyou 7’ were higher than ‘Tianyou 4’ under cold stress.

### Overview of sRNA sequencing in winter turnip rapes

**Fig. 2:**
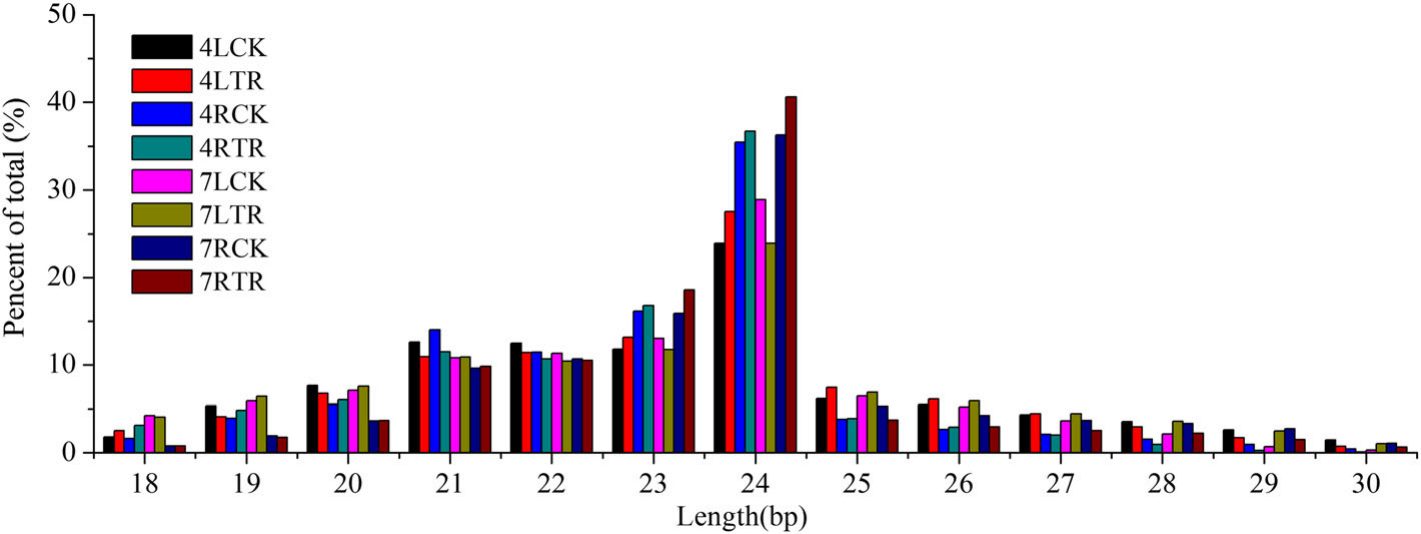
Sequence length distribution of sRNAs in winter turnip rape

To identify the miRNAs responding to cold stress, 16 sRNA libraries from eight samples (two biological replicates for each sample) were constructed. These libraries yielded a total of 228,117,863 raw reads by high-throughput sequencing (approximately 14.3 million raw reads per library). After removing the low-quality reads, sequences with adapter and polyA, sequences less than 18 nucleotides and more than 30, we obtained 214,293,155 clean reads and an average of 13,393,322 reads (93.97%) per library (Table 1). Of these reads, 181,942,519 clean reads were mapped to the database of miRBase [47] and Rfam (Rfam 12.2). The mapped sRNAs were annotated and classified into structural RNA (rRNA, tRNA, snRNA, snoRNA), known miRNAs (miRNAs in miRBase 21.0), precursor RNA, intergenic RNA, and unknown sRNAs (Table 2). Notably, approximately 2% of the reads were mapped to mature miRNAs in each sample (Table 2). The proportion of known miRNAs increased in 7RTR compared with 7RCK, implying that a number of miRNAs were induced in the roots of ‘Longyou 7’ under cold stress. The distribution of various size of sRNAs showed that 24 nt sRNAs are the most abundant, accounting for 23.9% (4LTR) to 40.6% (7RTR), following by 23 nt sRNAs (Fig. 2). The distribution is consistent with previous reports in various plants [48, 49]. To verify the results, we analyzed the Pearson correlation coefficients of the miRNA expression between the biological duplicates (Additional file 3: Figure S1), and the Pearson r value of miRNA expression were about 0.8.

**Table 1:** The statistics of sRNA-seq reads and mapped reads

Sample		Raw tag count	Clean tag count	Mapped tag
4LCK	4LCK1	14,616,241	13,450,762 (92.03%)	11,808,888 (87.79%)
	4LCK2	14,188,727	13,093,721 (92.28%)	11,354,540 (86.72%)
4LTR	4LTR1	13,857,570	13,091,406 (94.47%)	11,553,579 (88.25%)
	4LTR2	14,917,562	13,972,496 (93.66%)	12,378,849 (88.59%)
4RCK	4RCK1	14,030,718	13,581,849 (96.80%)	9,745,151 (71.75%)
	4RCK2	14,413,900	13,418,770 (93.1%)	11,029,802 (82.20%)
4RTR	4RTR1	14,204,427	13,435,279 (94.59%)	11,365,167 (84.59%)
	4RTR2	15,072,841	14,023,972 (93.04%)	11,923,337 (85.02%)
7LCK	7LCK1	14,317,284	13,327,929 (93.09%)	11,572,537 (86.83%)
	7LCK2	13,959,190	13,191,592 (94.50%)	11,629,503 (88.16%)
7LTR	7LTR1	14,658,533	13,284,854 (90.63%)	11,633,927 (87.57%)
	7LTR2	14,188,312	13,219,825 (93.17%)	11,649,344 (88.12%)
7RCK	7RCK1	13,871,471	13,214,641 (95.26%)	10,669,227 (80.74%)
	7RCK2	14,524,499	14,045,921 (96.71%)	11,739,632 (83.58%)
7RTR	7RTR1	14,264,729	13,425,669 (94.12%)	11,429,694 (85.13%)
	7RTR2	13,031,859	12,514,469 (96.03%)	10,459,342 (83.58%)
Total		228,117,863	214,293,155	181,942,519
Average		14,257,366	13,393,322 (93.97%)	11,371,407 (84.91%)

**Table 2:** Distribution of small RNAs among different categories in winter turnip rape

Category	Leaf				Root			
	4LCK	4LTR	7LCK	7LTR	4RCK	4RTR	7RCK	7RTR
Total	13,272,242 (100%)	13,531,951 (100%)	13,259,761 (100%)	13,252,340 (100%)	13,500,310 (100%)	13,729,626 (100%)	13,630,281 (100%)	12,970,069 (100%)
intergenic	10,863,143 (81.85%)	11,390,504 (84.17%)	11,144,905 (84.05%)	11,052,177 (83.40%)	9,907,946 (78.67%)	11,180,659 (81.43%)	10,725,549 (78.69%)	10,509,664 (81.03%)
snRNA	1626 (0.01%)	966 (0.01%)	1039 (0.01%)	1034 (0.01%)	2271 (0.02%)	1819 (0.01%)	1844 (0.01%)	2490 (0.02%)
rRNA	399,431 (3.01%)	336,734 (2.49%)	210,748 (1.59%)	349,445 (2.64%)	135,673 (0.77%)	109,499 (0.80%)	260,830 (1.91%)	175,824 (1.36%)
snoRNA	7396 (0.06%)	9558 (0.07%)	8350 (0.06%)	10,854 (0.08%)	7972 (0.05%)	8510 (0.06%)	8079 (0.06%)	16,507 (0.13%)
precursor	21,608 (0.16%)	10,156 (0.08%)	11,141 (0.08%)	13,346 (0.10%)	6579 (0.05%)	6436 (0.05%)	8380 (0.06%)	5120 (0.04%)
miRNA	317,989 (2.40%)	234,958 (1.74%)	241,114 (1.82%)	236,628 (1.79%)	339,170 (2.70%)	345,643 (2.52%)	218,823 (1.61%)	244,386 (1.88%)
tRNA	1219 (0.01%)	923 (0.01%)	543 (0.00%)	1496 (0.01%)	359 (0.00%)	125 (0.00%)	641 (0.00%)	230 (0.00%)
unmap	1,657,480 (12.49%)	1,546,089 (11.43%)	1,639,573 (12.37%)	1,584,846 (11.96%)	3,097,914 (17.73%)	2,075,386 (15.12%)	2,403,423 (17.63%)	2,014,395 (15.53%)

### Identification of conserved and novel miRNAs

**Fig. 3:**
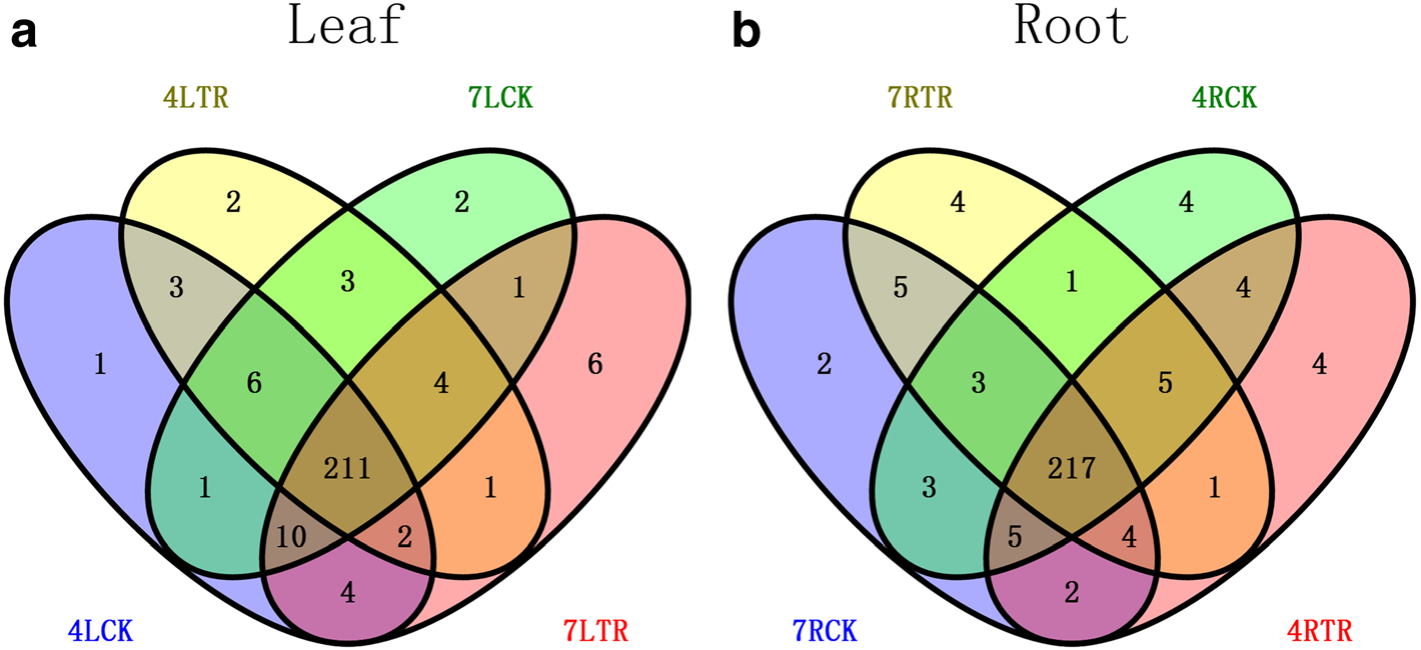
Analysis of identified conserve microRNAs in leaves and roots from two winter turnip rape varieties

Conserved miRNAs were identified by aligning against the miRBase 21.0 database. We obtained 269 conserved miRNAs belonging to 90 miRNA families from 16 libraries (Additional file 4: Table S3). And 96 members of them belonged to the known Bra-miRNAs, and 173 were similar to known miRNAs from other plant species. The result demonstrated that some conserved miRNAs in evolution also mediated the regulatory pathways of winter turnip rape. The expression profiles of the conserved miRNAs showed great variation in the amount of reads, which varied from 10,000 to less than 10 reads (Additional file 4: Table S3). Six miRNAs (miR158-3p, miR159a-1, miR162-3p, miR162a-3p, miR166, miR166a) presented high expression levels in all the samples. miR159a-1 was identified with the highest expression level, covering about 10,000 reads in 4LCK and 7LCK. In contrast, about 70 miRNAs were identified with less than 10 reads in all libraries. Besides, miR156 was identified as the largest miRNA family including 22 members, following by miR172 and miR319. Moreover, 222 and 226 known miRNAs were common between control and treatment from leaves of ‘Tianyou 4’ and ‘Longyou 7’, respectively (Fig. 3a). And 231 and 229 known miRNAs were common between control and treatment from roots of ‘Tianyou 4’ and ‘Longyou 7’, respectively (Fig. 3b), implying that known miRNAs are stable in different cultivars even under cold stress. The lengths of most of these miRNAs was 21 nt long, similar to those observed in previous reports [50, 51]. Characteristic information of all the conserved miRNA sequences was summarized in Additional file 4: Table S3.

**Fig. 4:**
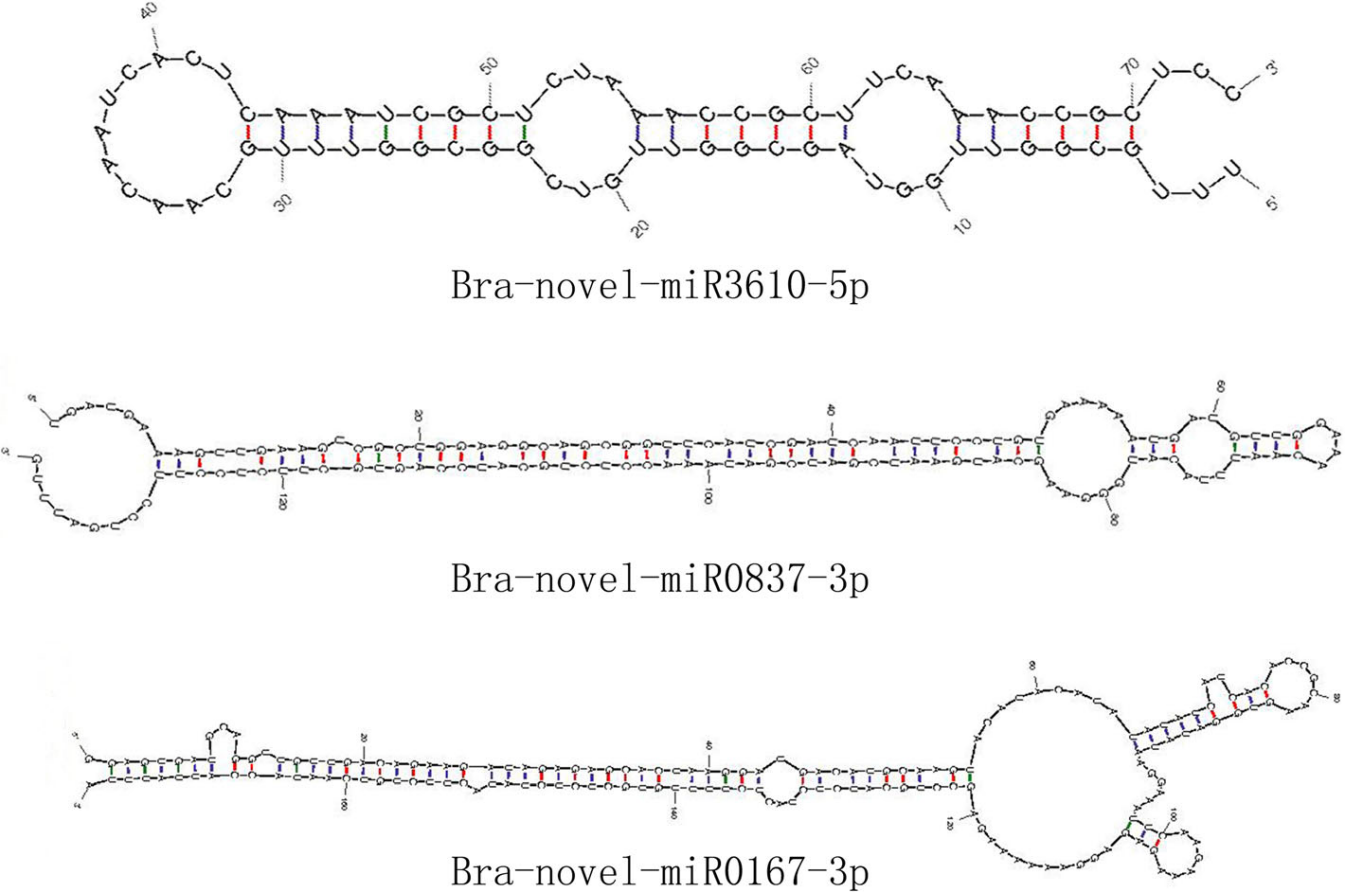
Secondary structures of differentially expressed novel miRNA precursors

For the identification of novel miRNA candidates, the unannotated sRNAs matched to the *B. rapa* genome sequence were analyzed by miRDeep2. All the novel miRNA sequences were named in the form of Bra-novel-miR-number. Generally, we identified 3538 novel miRNAs (Additional file 5: Table S4), and 84 of them have star sequences (Additional file 6: Table S5). The lengths of novel miRNAs with star sequences varied from 20 nt to 24 nt, with the majority of 21 nt in length. The mean length of these miRNA precursors was 148 nt, ranging from 65 to 373 nt (Additional file 6: Table S5). Most of the novel miRNAs were identified with relatively low read counts compared with conserved miRNAs. Only six novel miRNAs were identified with more than 100 reads, and most of the remaining novel miRNAs had less than 10 reads (Additional file 6: Table S5). To confirm whether these sRNAs were true miRNAs of winter turnip rape, we analyzed their hairpin structures with Mfold or RNAfold [52]. The minimum free energies of these pre-miRNA hairpin structures varied from − 170.6 kcal·mol^− 1^ to − 19.1 kcal·mol^− 1^ with an average of − 58.9 kcal·mol^− 1^ similar to those of other plant miRNA precursors [48]. The secondary structures of the three most abundant novel miRNAs (Bra-novel-miR0167-3p, Bra-novel-miR0837-3p, and Bra-novel-miR3610-5p) were predicted, indicating they can form typical stem-loop hairpins, and the folding free energy was − 52.99, − 54.80, and − 32.40 kcal/mol, respectively (Fig. 4).

### Differentially expressed miRNAs under cold stress

**Fig. 5:**
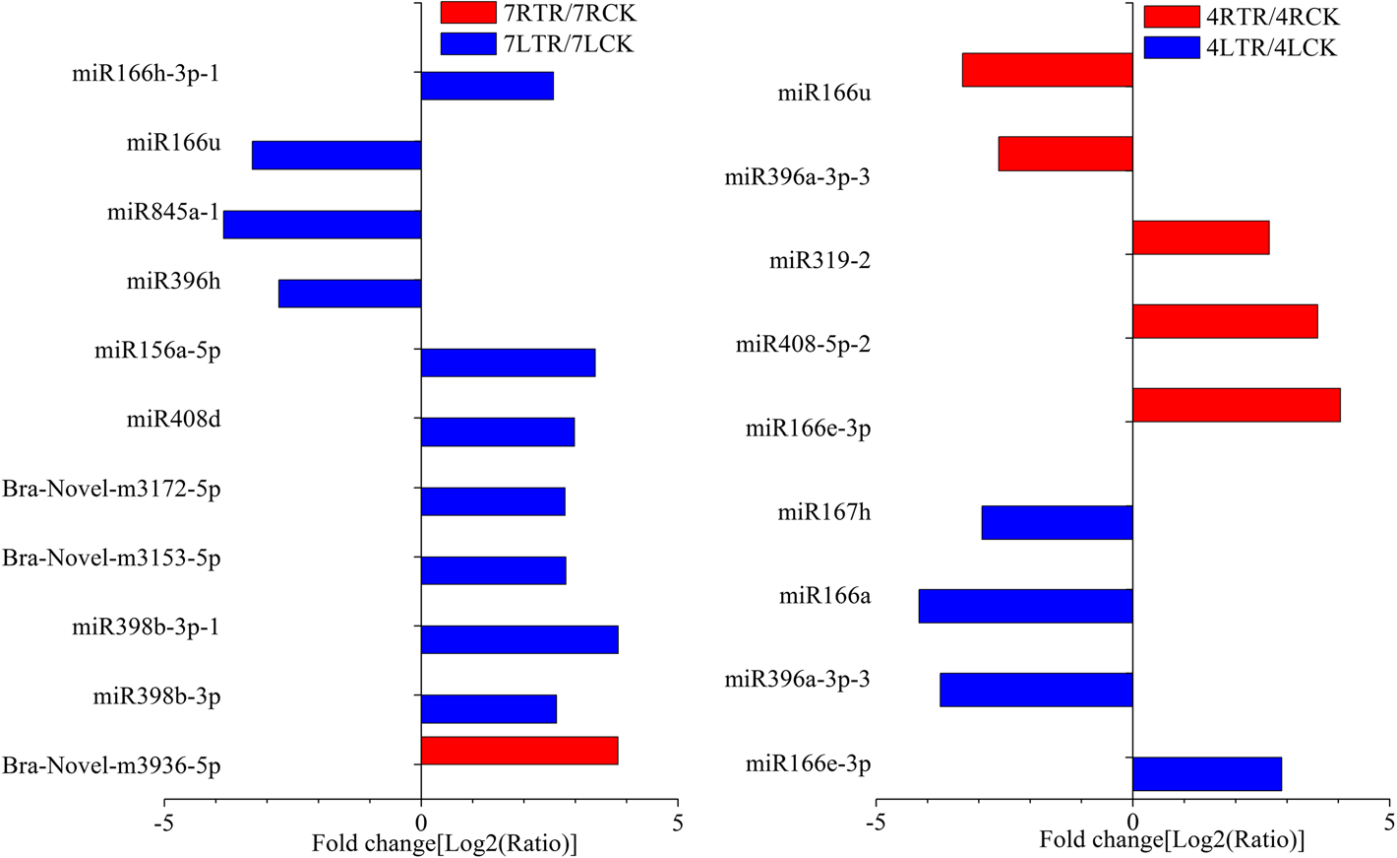
Differential expression of miRNAs under cold stress

The heat map presenting the expressional pattern of conserved and novel miRNAs in stressed and non-stressed leaves and roots of two *B. rapa* cultivars were shown in Additional file 7: Figure S2 and Additional file 8: Figure S3. Eleven differentially expressed miRNAs with more than two fold change were identified in ‘Longyou 7’ after cold stress, and nine differentially expressed miRNAs were identified in ‘Tianyou 4’ under cold stress (Additional file 9: Table S6). In detail, seven miRNAs (five known and two novel miRNAs) were up-regulated and three conserved miRNAs were down-regulated in leaves of ‘Longyou 7’ under cold stress. And Bra-novel-miR3936-5p was up-regulated in roots of ‘Longyou 7’ under cold stress. miR166e-3p was up regulated in leaves of ‘Tianyou 4’ under cold stress, while miR166a, miR167h and miR396-3p-3 were down-regulated in leaves of ‘Tianyou 4’ after cold stress. Three miRNAs (miR166e-3p, miR408-5p-2 and miR319–2) and two miRNAs (miR396a-3p-3 and miR166u) were up-regulated and down-regulated in stressed roots of ‘Tianyou 4’ compared with non-stressed roots of ‘Tianyou 4’, respectively (Fig. 5). We found two differential miRNAs (up-regulated miR166e-3p and down-regulated miR396a-3p-3) both in the leaves and roots of ‘Tianyou 4’ under cold stress.

### Comparative analysis of differentially expressed miRNAs between two varieties

The differentially expressed miRNAs between two varieties were compared. No significant difference was observed between non-stressed leaves of ‘Tianyou 4’ and ‘Longyou 7’, whereas miR166e-3p was significantly down-regulated and miR166h-3p was up-regulated in stressed leaves of ‘Longyou 7’compared with stressed leaves of ‘Tianyou 4′. We also identified two down-regulated miRNAs (miR319e and miR166m-2) between non-stressed roots of ‘Tianyou 4′ and ‘Longyou 7′, and eight differentially expressed miRNAs (Bra-novel-miR0040-3p, miR166e-3p, miR319e-1, miR319–2, and miR319a were down-regulated; Bra-novel-miR3153-5p, Bra-novel-m0894-3p and Bra-novel-m3936-5p were up-regulated) in stressed roots of ‘Longyou 7′ compared with stressed roots of ‘Tianyou 4′. We detected one co-expressed differential miRNA (miR166e-3p), which was down-regulated in both cold stressed leaves and roots of ‘Longyou 7′ compared with stressed organs of ‘Tianyou 4′ (Additional file 10: Table S7). By further analyzing these results, we found that all the differentially expressed known miRNAs of the two varieties belonged to miR166 and miR319 families. The identified members of miR319 were down-regulated in cold stressed leaf and root of ‘Longyou 7′ compared with ‘Tianyou 4′. However, members of the miR166 family showed differences in respect to their responses to cold stress. For example, miR166e-3p was down-regulated in the leaves and roots of ‘Longyou 7′ under cold stress, but miR166h-3p was up-regulated in the leaves. This result indicate that different expression pattern of miR166 members in different library might be related to their roles in the regulatory networks of cold stress.

### Target prediction of differentially expressed miRNAs and gene enrichment analysis

**Fig. 6:**
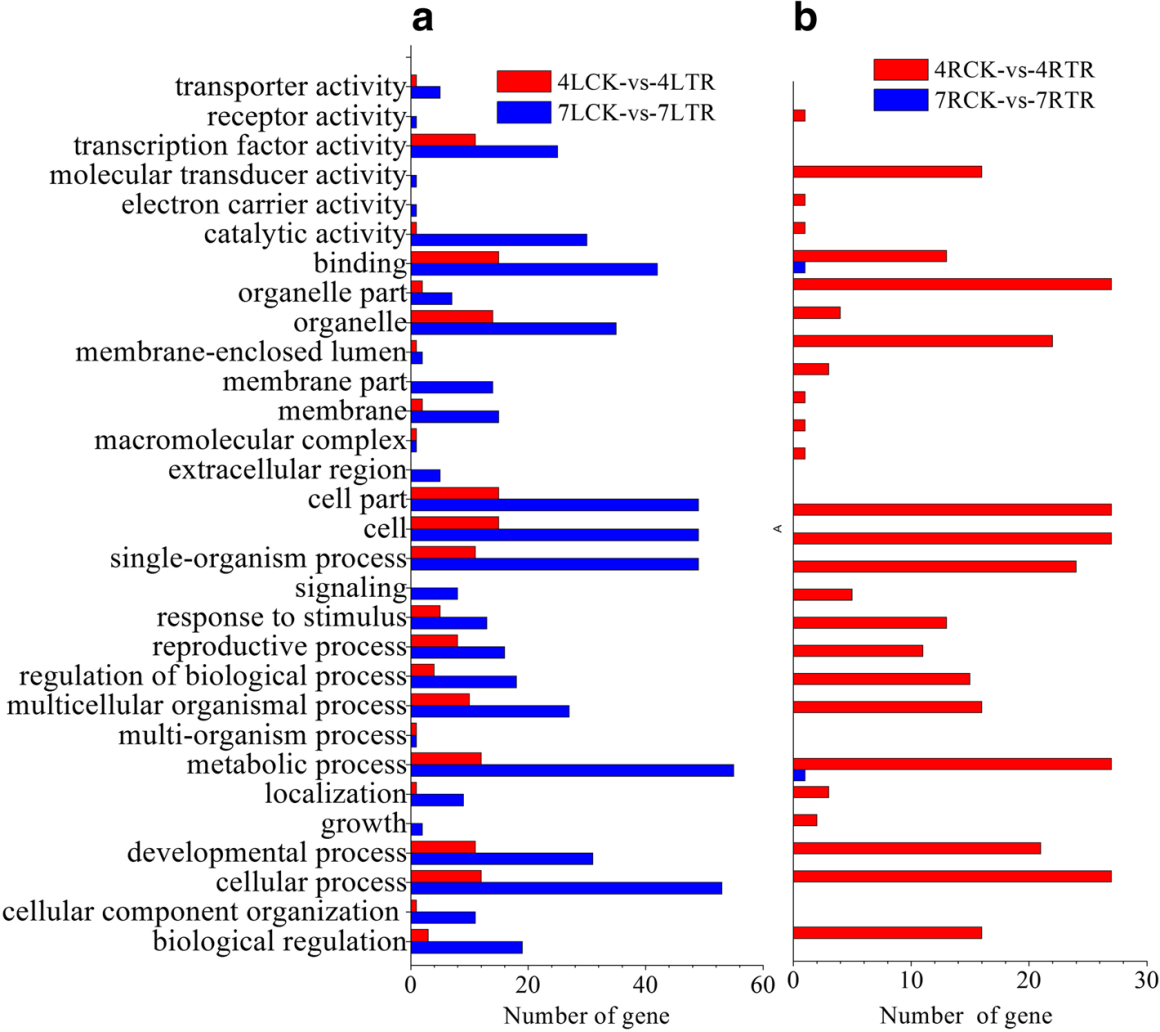
Gene Ontology (GO) categories and sub-categories of differentially expressed miRNA targets between the following samples: 7LCK versus7LTR, 4LCK versus 4LTR (A); 4RCK versus 4RTR, 7RCK versus 7RTR (B)

To acknowledge the potential functions of differentially expressed miRNAs, we used psRobot and TargetFinder to predict the target genes [53, 54]. All the target genes were subjected to GO enrichment analysis. A total of 211 genes for fifteen known and two novel miRNAs were predicted as potential miRNA targets (Additional file 11: Table S8). GO annotation of these target genes showed that some stress-related GO terms were enriched, including response to stimulus and signaling. The most enriched GO terms were metabolic process, cellular process, cell, cell part, and binding (Fig. 6). The target genes of the differentially expressed miRNAs (Bra-novel-miR3936-5p) in 7RCK and 7RTR were involved in the regulation of metabolic processes and binding (Fig. 6). The diverse functions of the predicted target genes suggested that the differentially expressed miRNAs may play important roles during plant response to cold stress.

### Validation of expression patterns of miRNAs and predicted target genes by qRT-PCR

**Fig. 7:**
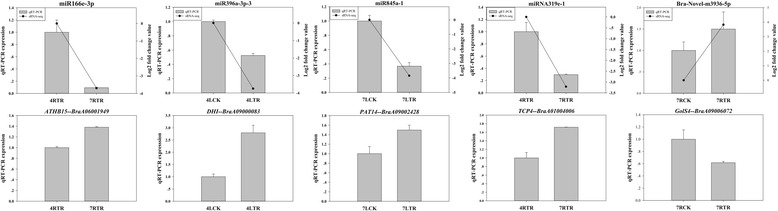
qRT-PCR analysis of differentially expressed miRNAs and the target genes

For the verification of the sequencing results, five miRNAs (miR166e-3p, miR396a-3p-3, miR845a-1, miR319e-1, and Bra-novel-miR3936-5p) and the corresponding target genes (BraA06001949, BraA09000083, BraA09002428, BraA01004006, and BraA09006072) were selected for qRT-PCR analysis. The results showed that the qRT-PCR data of the differentially expressed miRNAs were in accordance with the sequencing results, and the expressional changes of target genes were negatively correlated to differentially expressed miRNAs (Fig. 7).

## Discussion

Cold is a common stress facing by many plant species grown in northwest regions of China, which can induce cellular dehydration and membrane injury. *B. rapa* is a vegetative plant broadly grown in many regions, the selection and breeding of cold tolerance cultivars is of great importance to the vegetative supports in northwest regions. Plants enduring cold stress could adapt themselves with defense mechanisms, including physiological responses, adaptation in metabolic and signal transduction pathways, as well as gene expressional changes [55]. MDA is a product of lipid peroxidation, and the degree of membrane damage can be reflected by MDA level in the stressed tissue [56]. POD can maintain the balance of formation and elimination of ROS by scavenging excess ROS, which helps plants to endure stress conditions [29, 57]. Therefore, MDA and POD are important physiological indexes of stress responses. Under cold stress, the MDA content in leaves and POD activity in roots of ‘Longyou 7’ were higher than ‘Tianyou 4’, indicating cold stress led to more serious membrane damage in leaves of ‘Longyou 7’, but cold tolerance of ‘Longyou 7’ roots was stronger than ‘Tianyou 4’ under cold stress. These physiological changes under cold stress agreed to the previous reports [28, 58].

miRNAs as systemic regulators are involved in plant response to various abiotic stresses. Cold stress severely limits the geographic distribution of plant species by suppressing growth and development. Plants could adapt to cold stress by regulating gene expression. Moreover, 24 nt siRNAs can induce transcriptional gene silencing by DNA and histone modifications [59–62]. The relationship between miRNAs and cold response was reported in many plants [24, 25, 63, 64]. In winter turnip rape, the genome-wide expression profile of miRNAs in response to cold stress remains unreported. In the present study, millions of short reads were obtained from the sRNA libraries of two winter turnip rape cultivars under 20 °C and − 4 °C treatments. Similar to other plants, winter turnip rapes was enriched with 24 nt sRNAs [65, 66].The identified 269 conserved miRNAs (Additional file 4: Table S3) and 84 novel miRNAs (Additional file 6: Table S5) showed great variations in the amount of reads (from 1 to 94,237). The expression levels of novel miRNAs was relatively lower than that of conserved miRNAs, similar to the previous reports [67, 68]. The novel miRNAs could enrich the *B. rapa* miRNA database, which may also provide a foundation for cold-tolerant regulation of miRNA.

High-throughput sequencing platforms are widely used to identify miRNAs at a genome-wide scale. Our study was the first to identify differences between the miRNAs of the same variety under control and cold-stressed treatments. Cold stress triggered 10 differentially expressed miRNAs in the leaves of ‘Longyou 7’, but only one miRNA was up-regulated in the roots of ‘Longyou 7’. As previously reported, root tolerance to cold is very important for survival of ‘Longyou 7’ in winter [28]. Thus, we suspect the differentially expressed miRNA induced in roots of ‘Longyou 7’ might be valuable to its better survival rates compared with ‘Tianyou 4’. The aboveground part of ‘Longyou 7’ becomes more sensitive to cold stress, exhibiting poor growth and senescence [28]. Therefore, the number of differentially expressed miRNAs in the leaves of ‘Longyou 7’ was higher, which might be related to the solutions of ‘Longyou 7’ to alleviate cold induced injuries such as cytoplasmic damages caused by ice [55]. According to previous studies, minimal changes in miRNA expression can cause significant alterations in mRNA levels and related biological processes [69]. Thus, Bra-novel-miR3936-5p expressed in the roots may be a pivotal regulatory factor for the strong tolerance of ‘Longyou 7’ to cold stress. Furthermore, cold stress triggers four differentially expressed miRNAs in the leaves and five differentially expressed miRNAs in the roots of ‘Tianyou 4’. Of these miRNAs, two (miR166e-3p and miR396a-3p-3) were common in the leaves and roots of ‘Tianyou 4’, indicating that they were the most basic regulatory factors of cold stress in ‘Tianyou 4’. In summary, the differential expression of miRNAs implied the different response to cold stress in the two varieties.

In both varieties, no significant change was observed in the miRNAs expression of leaves, except for two miRNAs down-regulated in their roots under 20 °C. However, the number of differential miRNAs was increased under cold stress. Two differentially expressed miRNAs were found in the leaves and eight in the roots. These results indicated that the different expression levels of miRNAs under cold stress account for the different cold tolerance between two varieties. miRNAs with different expression pattern may provide a basis for the identification of molecular mechanism of cold tolerance in root of winter turnip rape. As reported, miRNAs regulate gene expression at the post-transcriptional level [70, 71]. The different expression levels of miRNAs between two varieties could attribute to explain the mechanisms of different cold tolerance. The root is the only overwintering organ of winter turnip rape, and its tolerance to cold stress is critical to their survival during winter. Under cold stress, eight miRNAs was differentially expressed between roots of ‘Longyou 7’ and roots of ‘Tianyou 4’, which might be related to the different root tolerance between two cultivars. We detected the expression of miR166e-3p was lower in the leaves and roots of ‘Longyou 7’ than in ‘Tianyou 4’, but its family member miR166h-3p was highly expressed in the leaves of ‘Longyou 7’ than in ‘Tianyou 4’, indicating that miR166 plays important role in the regulation of plant response to cold.

miRNAs participate in many biological processes through regulating the expression of target genes. Thus, identify the putative function of the target genes is important to acknowledge the miRNA-mediated mechanisms. We predicted 211 target genes with diverse functions for 15 known miRNAs and two novel miRNAs using bioinformatics approach (Additional file 11: Table S8). The majority of the predicted target genes encoded transcription factors, such as ATHB, MYB, GAMYB, TCP, bHLH transcription factor, Argonaute, and SQUAMOSA promoter binding proteins. Furthermore, other target genes, encoding glucosidase, glycylpeptide transferase, laccase, acyl-lipid desaturase, and galactinol synthase, are involved in various physiological and metabolic processes. Many other biological processes, including development, signal transduction, metabolism, and responses to stress are also regulated by miRNA.

In our study, the members of miR156, miR166 miR396 and miR845 family showed different expression pattern under cold stress (Additional file 9: Table S6). *Squamosa-promoter binding protein-like* (*SPL*) is the target gene of miR156, which has been reported with function in regulating leaf development [72, 73]. We found miRNA156a-5p was up-regulated in leaves of ‘Longyou 7’ under cold stress, whereas no difference of miR156a expression was observed in roots (Additional file 9: Table S6). The same results has been reported in barley under dehydration stress [74]. miR156 was up-regulated in *Arabidopsis* seedlings under salt stress [24]. The expressional changes of miR156a in leaves of ‘Longyou 7’ indicate it might be responsive to cold stress. Freeze injury can lead to plant dehydration and damage intracellular membrane system [75, 76]. Thus, we speculate that up-regulation of miRNA156a-5p might be resulted from the leaf dehydration and intracellular membrane damages in ‘Longyou 7’ under cold stress. And the aboveground of ‘Longyou 7’ could be influenced by the expressional changes of miR156 and the target genes, whereas there is no effect on roots.

miR845 has been reported with functions in regulating abiotic stress [77], but the mechanism of its role remains unclear. In our study, miR845a-1 targets a gene encoding probable protein S-acyltransferase 14 (PAT14) that involved in leaf senescence, and was over-expressed in senescent leaves of *Arabidopsis* [78]. Leaf senescence is a critical process in plant development and stress responses [79]. miR845a-1 was down-regulated in leaves of ‘Longyou 7’ under cold stress, and the target gene *PAT14* was up-regulated (Fig. 7), which might be responsible for the earlier leaf senescence in ‘Longyou 7’ under cold stress.

Transcription factor *TCP4-like* (*TCP4*) is the main target gene of miR319. A recent research found miR319-mediated *TCP4* controlled secondary cell wall formation by activating the expression of a downstream *VND7* transcriptional network in *A. thaliana*. And the overexpression of *TCP4* could significantly increase cell wall thickness, promote the formation of xylem vessel and deposition of lignin and cellulose [80]. Increase of cell wall thickness and lignification could increase cold tolerance in plants [81]. Under cold stress, we found three miR319 members (miR319e-1, miR319a and miR319–2) was down-regulated in roots of ‘Longyou 7’ compared with ‘Tianyou 4’, and expression of *TCP4* was increased in roots of ‘Longyou 7’ (Fig. 7). These changes might lead to thicker cell wall in roots of ‘Longyou 7’ than that of ‘Tianyou 4’, which could be helpful to the cold tolerance of ‘Longyou 7’.

Homeobox-leucine zipper protein ATHB15, encoded by the target gene of miR166e-3p, is important to the synthesis of plant cell wall and cellulose [82]. In our study, the down-regulation of miR166e-3p and up-regulation of *ATHB-15* in roots of ‘Longyou 7’ compared with ‘Tianyou 4’ under cold stress was identified (Fig. 7), which might improve cold tolerance in ‘Longyou7’ by increasing cell wall thickness and cellulose accumulation.

Most *galactinol synthase* (*GolS*) genes were reported to be induced by abiotic stresses, which can increase amounts of galactinol and raffinose to improve stress tolerance of plants [83–86]. In this study, we found Bra-novel-miR3936-5p targets *GolS4* (BraA09006072), which could encode galactinol synthase and take part in the biosynthesis of raffinose oligosaccharides (ROFs) (Additional file 11: Table S8). Interestingly, Bra-novel-miR3936-5p was up-regulated in roots of ‘Longyou 7’ under cold stress compared with ‘Tianyou 4’, which led to the down-regulation of *Gols4* (BraA09006072). In *Populus trichocarpa, PtrGolS4* was down-regulated under cold stress, whereas the expression of some *GolS* homologous was induced, indicating *PtrGolS4* with no contribution to the accumulation of galactinol and raffinose under cold stress [87]. Feng et al. (2017) found that *VvGolS4* could participate in H_2_S-mediated pathways that was responsive to temperature changes [88]. The expressional changes of Bra-novel-miR3936-5p and *GolS4* in ‘Longyou 7’ might be related to cold stress responses involving some metabolic pathways. Bra-novel-miR3936-5p was confirmed as a cold stress-induced up-regulated miRNA in roots of ‘Longyou 7’, this is valuable for the dissection of cold tolerance in ‘Longyou 7’ compared with ‘Tianyou 4’.

Overall, we identified and compared cold-responsive miRNAs in the leaves and roots of two winter turnip rape varieties using high-throughput sequencing, which were selected for stem-loop qRT-PCR validation. The data could lay a base for the identification of miRNAs related to cold tolerance. Prediction and functional annotation of target genes were carried out to known the putative function of miRNAs in cold stress responses. However, whether these differentially expressed miRNAs are true regulators in cold tolerance of ‘Longyou 7’, especially in the roots, need more experimental validations. For instance, genetic transformation of precursors of these candidate miRNAs into ‘Tianyou 4’ to validate its molecular function, accompany cold-tolerant phenotypes analysis in a segregating population with different miRNA expression levels, would help to confirm the molecular function of these miRNAs in regulating cold stress responses.

## Conclusions

In the present study, small RNA libraries of cold stressed and non-stressed leaves and roots of two winter turnip rape varieties were constructed separately, including two replicates for each sample. As a result, a set of conserved and novel miRNAs were identified by high throughput sequencing. The differentially expressed miRNAs were screened to find stress responsive miRNAs by comparing different organs and cultivars, as well as different treatments (cold stressed and non-stressed). The results indicated many candidate miRNAs and target genes with potential functions in regulating cold tolerance in ‘Longyou 7’, which are valuable for dissecting post-transcriptional and transcriptional regulations of cold responses, and *B. rapa* improvement in cold regions in the world.

## Additional files

Additional file 1: **Table S1.** Primers for qRT-PCR analysis of differentially expressed miRNAs (DOCX 14 kb)

Additional file 2: **Table S2.** Primers for qRT-PCR analysis of target genes of differentially expressed miRNAs (DOC 32 kb)

Additional file 3: **Figure S1.** The correlation analysis of miRNA expression between two biological duplicates (JPEG 3923 kb)

Additional file 4: **Table S3.** The conserved miRNAs identified in winter turnip rape (XLS 76 kb)

Additional file 5: **Table S4.** The novel miRNAs identified in winter turnip rape (XLS 1404 kb)

Additional file 6: **Table S5.** The novel miRNAs identified in winter turnip rape with star sequences (XLS 58 kb)

Additional file 7: **Figure S2.** Heat map of conserved miRNAs identified in winter turnip rape (JPEG 1334 kb)

Additional file 8: **Figure S3.** Heat map of novel miRNAs identified in winter turnip rape (JPEG 526 kb)

Additional file 9: **Table S6.** Differentially expressed miRNAs between cold-stressed and non-stressed samples of two winter turnip rape varieties (DOC 53 kb)

Additional file 10: **Table S7.** Differentially expressed miRNAs between two varieties (DOC 41 kb)

Additional file 11: **Table S8.** Targets prediction of all the sequenced miRNAs (XLS 57 kb)

## Abbreviations

ATHB15: Homeobox-leucine zipper protein 15
CK: Control
GO: Gene ontology
GolS: Galactinol synthase
MDA: Malondialdehyde
miRNA: microRNA
nt: Nucleotide
PAT14: Probable protein S-acyltransferase14
PCR: Polymerase chain reaction
POD: Peroxidase
qRT-PCR: Quantitative real time polymerase chain reaction
RISCs: RNA-induced silencing complexes
ROS: Reactive oxygen species
rRNA: Ribosomal RNA
snoRNA: Small nucleolar RNA
snRNA: Small nuclear RNA
sRNA: Small RNA
TCP4: Transcription factor TCP4-like
TF: Transcription factor
TPM: Transcripts per kilobase million
TR: Treatment
tRNA: Transfer RNA
UTR: Untranslated region
WEGO: Web gene ontology annotation plot
