# Supplementary material for: Identification of cold stress responsive microRNAs in two winter turnip rape (Brassica rapa L.) by high throughput sequencing
Source: BMC Plant Biol. 2018 Mar 27;18:52. doi: 10.1186/s12870-018-1242-4 (PMC5870505; doi:10.1186/s12870-018-1242-4)
Supplement: Supplementary file 1 — Table S1. Primers for qRT-PCR analysis of differentially expressed miRNAs (DOCX 14 kb) [file 12870_2018_1242_MOESM1_ESM.docx]

**Table S1 Primers for qRT-PCR analysis of differentially expressed miRNAs**

| **miRNA** | **Primer Sequences (5'--3')** | |
| --- | --- | --- |
|  | **RT primer** | **primer F** |
| miR166e-3p | GTCGTATCCAGTGCAGGGTCCGAGGTATTCGCACTGGATACGACGGGAAT | CTCGGACCAGGCTTCATTCC |
| miR396a-3p-3 | GTCGTATCCAGTGCAGGGTCCGAGGTATTCGCACTGGATACGACCTTCCC | TTCAATAAAGCTGTGGGAAGG |
| miR845a-1 | GTCGTATCCAGTGCAGGGTCCGAGGTATTCGCACTGGATACGACCATCAA | CGGCTCTGATACCAATTGAT |
| miR319e-1 | GTCGTATCCAGTGCAGGGTCCGAGGTATTCGCACTGGATACGACAGGAGC | CAGCTTGGACTGAAGGGAGC |
| Bra-novel-miR3936-5p | GTCGTATCCAGTGCAGGGTCCGAGGTATTCGCACTGGATACGACCGATCC | ATCTGGCGGATCAAGTGGAT |
| universal primer | CAGTGCAGGGTCCGAGGTAT |  |
| U6 | (U6-R)ACGCTCCACAGTCTGATGTAGC | ACTTGGTCCTGCTTCGAGATGG |
