# Supplementary material for: Identification of cold stress responsive microRNAs in two winter turnip rape (Brassica rapa L.) by high throughput sequencing
Source: BMC Plant Biol. 2018 Mar 27;18:52. doi: 10.1186/s12870-018-1242-4 (PMC5870505; doi:10.1186/s12870-018-1242-4)
Supplement: Supplementary file 2 — Table S2. Primers for qRT-PCR analysis of target genes of differentially expressed miRNAs (DOC 32 kb) [file 12870_2018_1242_MOESM2_ESM.doc]

**Table S2 Primers for qRT-PCR analysis of target genes of differentially expressed miRNAs**

| **Targets** | **Primer Sequences (5'--3')** | |
| --- | --- | --- |
|  | **primer F** | **primer R** |
| *β-actin* | CACTTCTCCTCCTTCTTTGG | GTAGGCATCCTTCTGGTTCA |
| BraA06001949 | GACAATAGCGTTTGAGTTT | GTTTGGGCTTCAGGAGTA |
| BraA09000083 | GACATGCCGCCATCTATC | AAGCCTTGTCTTCTACACTTGA |
| BraA09002428 | TGCGATGGAGTCTGTGAA | TGAACCGAACCTTGTGAA |
| BraA01004006 | GCCCATGATCCGTGCTTG | GACCCTGAAACCGTGCTG |
| BraA09006072 | TGGCAGGTAATGGAGACT | CACATCAGGCATCACAGC |
