# Supplementary figures and images for: Identification of cold stress responsive microRNAs in two winter turnip rape (Brassica rapa L.) by high throughput sequencing
Source: BMC Plant Biol. 2018 Mar 27;18:52. doi: 10.1186/s12870-018-1242-4 (PMC5870505; doi:10.1186/s12870-018-1242-4)

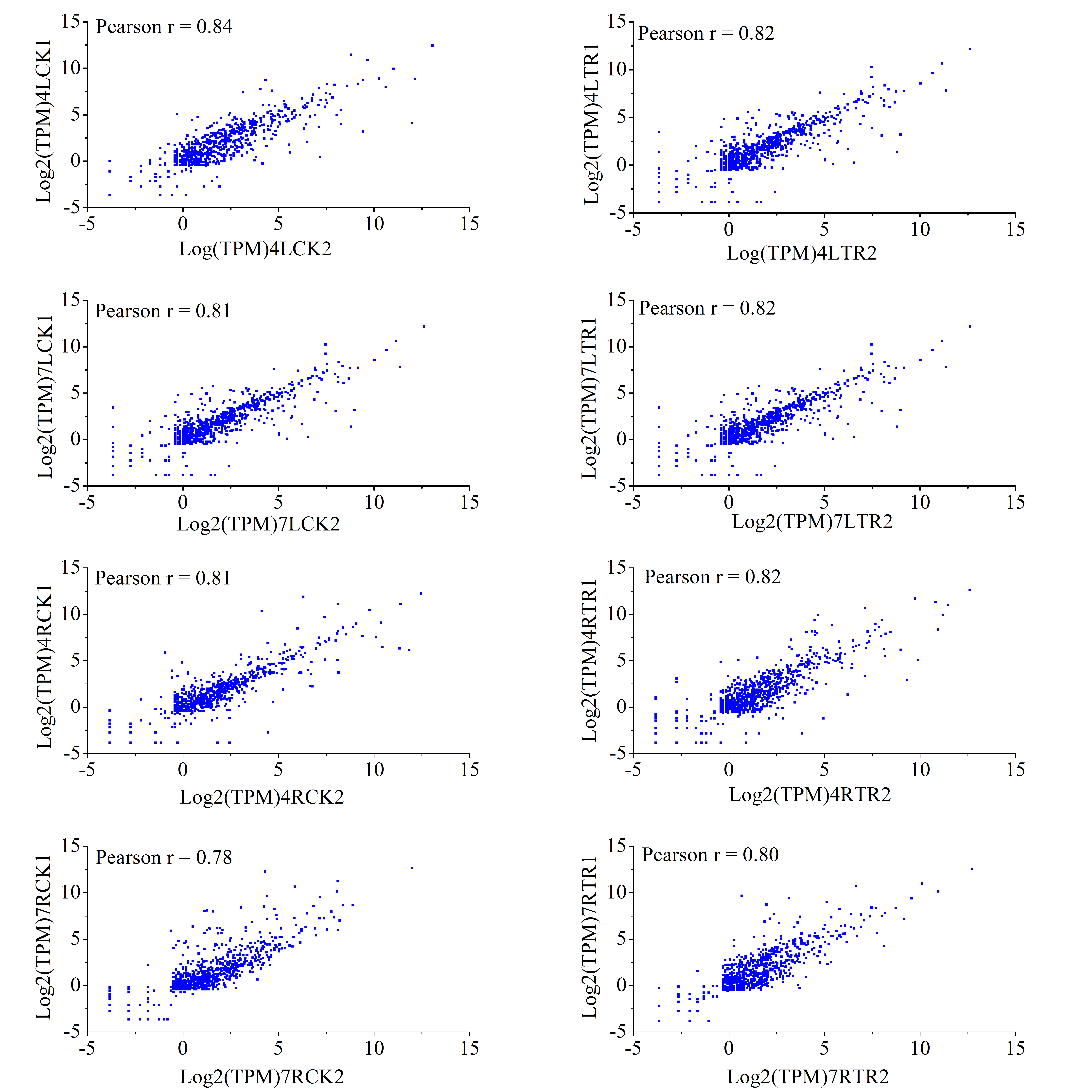

Supplement: Supplementary file 3 — Figure S1. The correlation analysis of miRNA expression between two biological duplicates (JPEG 3923 kb) [file 12870_2018_1242_MOESM3_ESM.jpg]

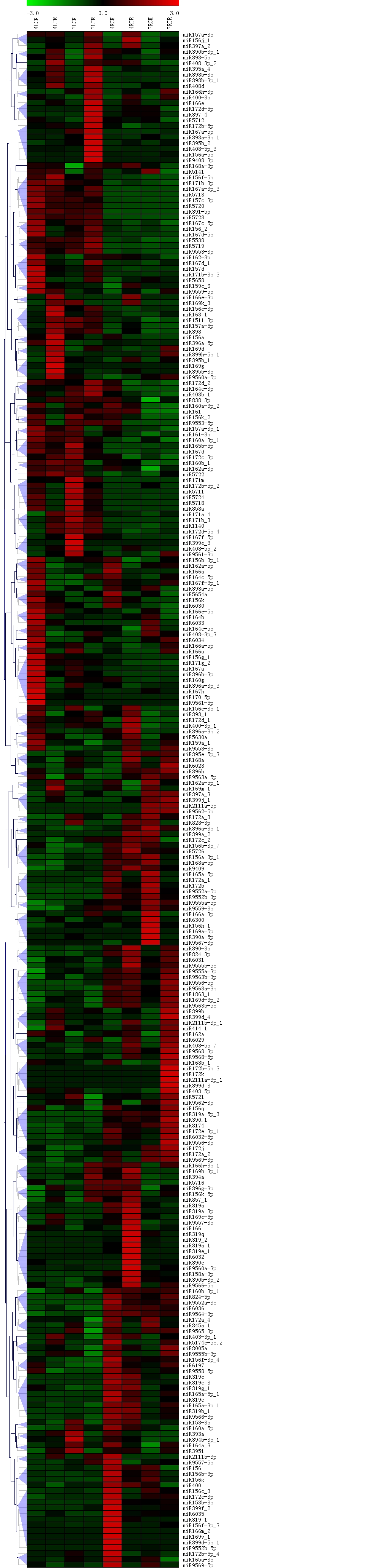

Supplement: Supplementary file 7 — Figure S2. Heat map of conserved miRNAs identified in winter turnip rape (JPEG 1334 kb) [file 12870_2018_1242_MOESM7_ESM.jpg]

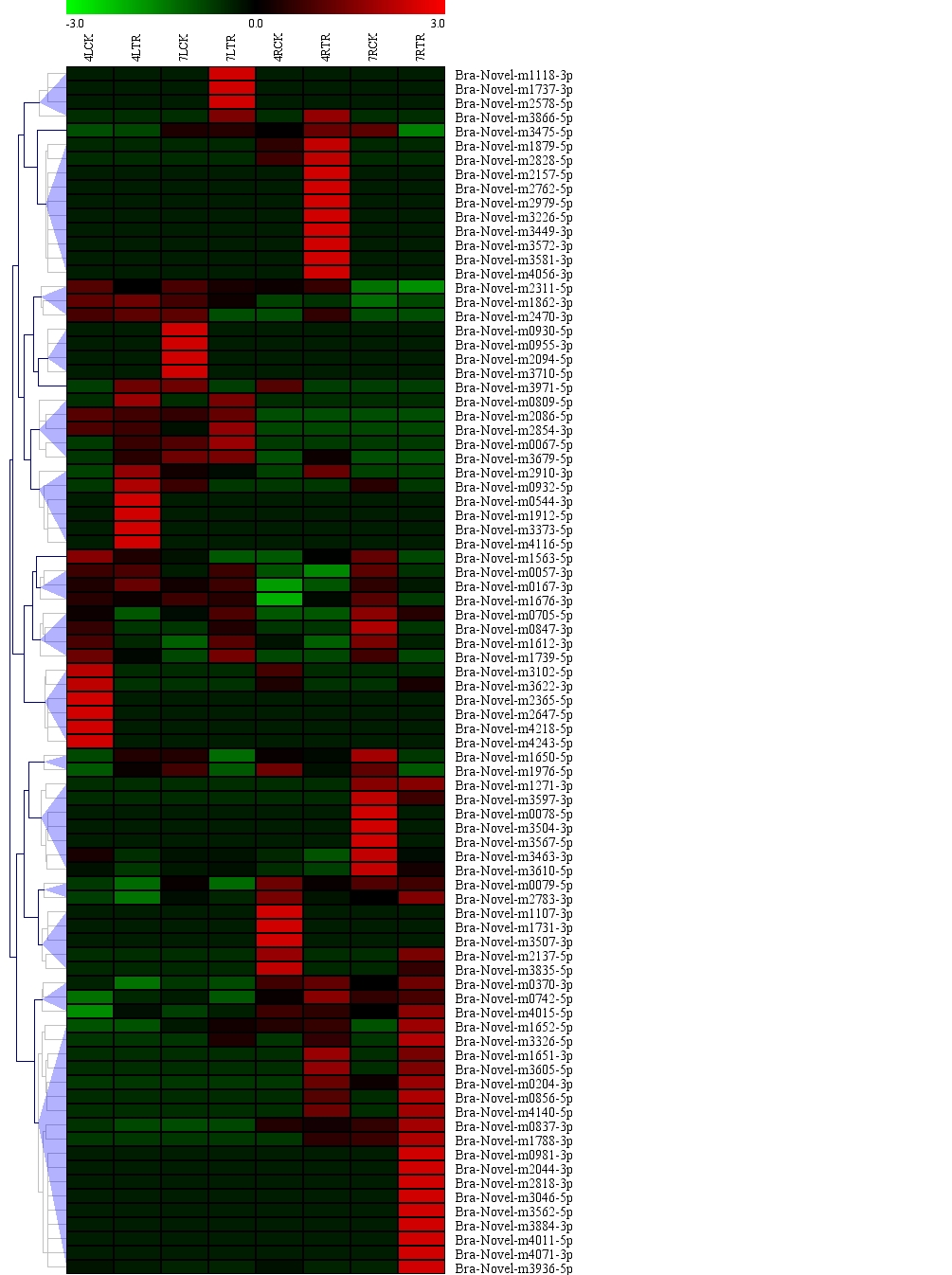

Supplement: Supplementary file 8 — Figure S3. Heat map of novel miRNAs identified in winter turnip rape (JPEG 526 kb) [file 12870_2018_1242_MOESM8_ESM.jpg]
