# Supplementary material for: Identification of cold stress responsive microRNAs in two winter turnip rape (Brassica rapa L.) by high throughput sequencing
Source: BMC Plant Biol. 2018 Mar 27;18:52. doi: 10.1186/s12870-018-1242-4 (PMC5870505; doi:10.1186/s12870-018-1242-4)
Supplement: Supplementary file 9 — Table S6. Differentially expressed miRNAs between cold-stressed and non-stressed samples of two winter turnip rape varieties (DOC 53 kb) [file 12870_2018_1242_MOESM9_ESM.doc]

| Different  organ | Type | miRNA id | Expression(CK) | Expression(TR) | log2FoldChange  (TR/CK) | Pvalue | Padj | Up/Down-  Regulation |
| --- | --- | --- | --- | --- | --- | --- | --- | --- |
| Leaves | 4LCK-vs  -4LTR | miR166e-3p | 1460 | 27304.48 | 2.897711477 | 0.000147224 | 0.070998881 | Up |
|  |  | miR396a-3p_3 | 11227.3 | 390.16 | -3.750241312 | 3.58E-07 | 0.000344845 | Down |
|  |  | miR166a | 22046.69 | 368.59 | -4.163340105 | 3.61E-08 | 6.96E-05 | Down |
|  |  | miR167h | 566.81 | 30 | -2.937614304 | 0.000139875 | 0.070998881 | Down |
|  |  |  |  |  |  |  |  |  |
|  | 7LCK-vs  -7LTR | miR166h-3p_1 | 232.84 | 2277.88 | 2.574736067 | 9.18E-05 | 0.028603306 | Up |
|  |  | miR398b-3p | 690.45 | 7770.29 | 2.633241558 | 0.000128913 | 0.034419737 | Up |
|  |  | miR398b-3p_1 | 16.32 | 712.96 | 3.836592099 | 8.09E-08 | 8.82E-05 | Up |
|  |  | Bra-Novel-m3153-5p | 0 | 150.59 | 2.815614711 | 0.000399789 | 0.080970197 | Up |
|  |  | Bra-Novel-m3172-5p | 0 | 101.84 | 2.798630396 | 0.000433227 | 0.080970197 | Up |
|  |  | miR408d | 166.17 | 2790.65 | 2.9825617 | 2.87E-05 | 0.010722304 | Up |
|  |  | miR156a-5p | 10.55 | 896.47 | 3.391090179 | 1.38E-05 | 0.006450826 | Up |
|  |  | miR396h | 227.04 | 11.03 | -2.772000055 | 0.000257785 | 0.060224938 | Down |
|  |  | miR845a_1 | 5771.47 | 117.88 | -3.846985295 | 9.44E-08 | 8.82E-05 | Down |
|  |  | miR166u | 24316.12 | 1226.2 | -3.290366092 | 1.36E-06 | 0.000844242 | Down |
| Roots | 4RCK-vs  -4RTR | miR166e-3p | 537.95 | 22919.09 | 4.038088413 | 5.16E-08 | 0.000119423 | Up |
|  |  | miR408-5p_2 | 13.55 | 689.81 | 3.596135036 | 4.99E-06 | 0.004472425 | Up |
|  |  | miR319_2 | 315.92 | 3761.54 | 2.653133952 | 0.000137156 | 0.079413303 | Up |
|  |  | miR396a-3p_3 | 3321.73 | 454.77 | -2.612222624 | 0.000203697 | 0.0943526 | Down |
|  |  | miR166u | 8506.61 | 453.72 | -3.315199384 | 5.79E-06 | 0.004472425 | Down |
|  | 7RCK-vs  -7RTR | Bra-Novel-m3936-5p | 0 | 232.72 | 3.828934841 | 7.07E-06 | 0.015717323 | Up |

**Table S6 Differentially expressed miRNAs between cold-stressed and non-stressed samples of two winter turnip rape varieties**
