# Supplementary material for: Identification of cold stress responsive microRNAs in two winter turnip rape (Brassica rapa L.) by high throughput sequencing
Source: BMC Plant Biol. 2018 Mar 27;18:52. doi: 10.1186/s12870-018-1242-4 (PMC5870505; doi:10.1186/s12870-018-1242-4)
Supplement: Supplementary file 10 — Table S7. Differentially expressed miRNAs between two varieties (DOC 41 kb) [file 12870_2018_1242_MOESM10_ESM.doc]

**Table S7 D**ifferentially expressed miRNAs between two varieties

| 4LTR-vs  -7LTR | miRNA id | Expression (Tianyou4) | Expression (Longyou7) | log2FoldChange (Longyou7/Tianyou4) | Pvalue | Padj | Up/Down-  Regulation |
| --- | --- | --- | --- | --- | --- | --- | --- |
|  | miR166e-3p | 27190.84 | 200.09 | -5.015842364 | 3.75E-13 | 7.07E-10 | Down |
|  | miR166h-3p | 177.75 | 7627.44 | 4.0293086 | 9.02E-09 | 8.49E-06 | Up |
| 4RCK-vs  -7RCK | miR319e | 12702.38 | 131.06 | -4.305272918 | 1.68E-07 | 0.000351698 | Down |
|  | miR166m_2 | 26169.17 | 207.13 | -3.698282412 | 2.35E-05 | 0.02463724 | Down |
|  |  |  |  |  |  |  |  |
| 4RTR-vs  -7RTR | Bra-Novel-m3153-5p | 0 | 98.47 | 3.703164134 | 6.37E-05 | 0.022503068 | Up |
|  | Bra-Novel-m0894-3p | 0 | 142.91 | 4.428428224 | 9.78E-07 | 0.0008055 | Up |
|  | Bra-Novel-m3936-5p | 0 | 220.61 | 4.736775165 | 1.44E-07 | 0.00017774 | Up |
|  | Bra-Novel-m0040-3p | 158.51 | 0 | -4.193804548 | 4.11E-06 | 0.002029631 | Down |
|  | miR166e-3p | 24027.69 | 695.77 | -3.70010108 | 1.64E-06 | 0.001016169 | Down |
|  | miR319e_1 | 1268.61 | 35.52 | -3.213733574 | 0.000262016 | 0.080963032 | Down |
|  | miR319a | 625.8 | 21.36 | -3.500434361 | 1.63E-05 | 0.006720276 | Down |
|  | miR319_2 | 3781.57 | 9.9 | -6.19283524 | 7.96E-16 | 1.97E-12 | Down |
